# MicroRNA-582-5p promotes triple-negative breast cancer invasion and metastasis by antagonizing CMTM8

**DOI:** 10.1080/21655979.2021.2000741

**Published:** 2021-12-02

**Authors:** Xue Zeng, Xinchi Ma, Hong Guo, Linlin Wei, Yaotian Zhang, Chaonan Sun, Ning Han, Shichen Sun, Na Zhang

**Affiliations:** Department of Radiation Oncology, Cancer Hospital of China Medical University, Liaoning Cancer Hospital & Institute, Shenyang, China

**Keywords:** Breast cancer, CMTM8, metastasis, miR-582-5p, invasion

## Abstract

Triple-negative breast cancer (TNBC) commonly have aggressive properties. microRNA-582-5p (miR-582-5p) modulates the progression of several cancers. Yet, the role of miR-582-5p in TNBC progression is undetermined. In the current study, we investigated miR-582-5p expression levels and clinical significance in TNBC. The impact of miR-582-5p modulation on the biological behaviors of TNBC cells were measured. The downstream gene(s) regulated by miR-582-5p in TNBC was explored. We showed that compared to adjacent normal breast tissues, the miR-582-5p level was elevated in TNBC samples. The upregulation of miR-582-5p correlated with lymph node metastasis. Overexpression of miR-582-5p enhanced TNBC cell migration and invasion, whereas knockdown of miR-582-5p had an adverse impact on aggressive phenotype. In vivo xenograft mouse studies demonstrated that miR-582-5p overexpression accelerated TNBC growth and metastasis. Mechanistically, miR-582-5p selectively inhibited CMTM8, leading to a reduction of CMTM8 expression. CMTM8 showed suppressive effects on TNBC cell migration and invasion. Rescue experiments revealed that overexpression of CMTM8 impaired miR-582-5p-induced migration and invasion in TNBC cells. Overall, our data uncover an oncogenic role for miR-582-5p in TNBC metastasis through inhibition of CMTM8. We suggest miR-582-5p as a promising target for managing TNBC.

## Introduction

Triple-negative breast cancer (TNBC), which lacks the expression of HER2 or hormone receptors, is a lethal subtype of breast cancer [1]. TNBC accounts for 15–20% of all breast cancers, and commonly has a worse prognosis than other breast cancer types [2,3]. The invasive and metastatic phenotype of cancer cells is responsible for TNBC-related poor prognosis. However, there is no effective systematic therapy available for metastatic TNBC. Therefore, it is important to uncover the molecular mechanisms involved in TNBC metastasis.

CMTM8 is a transmembrane protein ubiquitously expressed in normal tissues [4]. A number of cancers such as liver, lung, colon, rectum, esophagus, stomach, and bladder cancers exhibited downregulation of CMTM8 [5–7]. Downregulation of CMTM8 contributes to epithelial-to-mesenchymal transition (EMT) and increases invasive ability in hepatocellular carcinoma cells [6]. Another study has reported that overexpression of CMTM8 suppresses cell proliferation and invasion in bladder cancer cells [7], indicating the involvement of CMTM8 in tumor metastasis.

MicroRNAs (miRs) are small noncoding RNAs that can bind to the 3′‐untranslated region (3′‐UTR) of target mRNAs, consequently causing mRNA degradation or translational repression [8,9]. MiRs affect a wide range of cellular behaviors, including cell proliferation, inflammation, infection, migration, differentiation, and tumorigenesis [10,11]. MiR-582-5p exerts tumor-suppressive effects in bladder cancer [12], hepatocellular carcinoma [13], colorectal carcinoma [14], gastric cancer [15], and prostate cancer [16]. However, this miR does not always elicit tumor suppression. For instance, Floyd et al [17] reported that miR-582-5p enhances human glioblastoma stem cell survival. Maeno et al [18] reported that in the setting of androgen deprivation, overexpression of miR-582-5p leads to an enhancement of prostate cancer growth. Despite these studies, relatively little is explored regarding the expression and function of miR-582-5p in breast cancer, especially TNBC.

We hypothesized that miR-582-5p might also play an important role in TNBC progression. Hence, in the present study, we determined the expression of miR-582-5p in TNBC specimens and checked its relationship with clinicopathological parameters of TNBC patients. The ability of miR-582-5p in regulating TNBC cell invasion and metastasis was investigated. Additionally, we identified the direct target gene(s) that mediates the action of miR-582-5p in TNBC.

## Materials and methods

### Patients and tissue specimens

We collected 68 paired TNBC and adjacent normal tissues from TNBC patients who underwent radical mastectomy or breast conserving surgery. Those patients did not receive any anticancer treatment prior to tumor resection. All tissue specimens were frozen immediately in liquid nitrogen and stored at −80°C.

### Cell culture

TNBC cell lines (MDA-MB-436, BT-549, MDA-MB-468, and MDA-MB-231) were cultured in RPMI 1640 medium supplemented with 10% fetal bovine serum (FBS; Invitrogen, Grand Island, NY, USA). MCF-10A mammary epithelial cells were cultured in DMEM/F12 supplemented with 5% donor horse serum (Invitrogen), 10 μg/mL insulin, and 20 ng/mL epidermal growth factor (Peprotech, Rocky Hill, NJ, USA).

### Quantitative real-time PCR (qPCR) analysis

Trizol reagent (Invitrogen) was used for total RNA isolation. Quantification of miR-582-5p was performed as described previously [14]. cDNA was reverse-transcribed from RNA using the miRNA Reverse Transcription kit (Vazyme Biotech, Nanjing, China). The primers were as follows: miR-582-5p, forward: 5′-GCACACATTGAAGAGGACAGAC-3′, reverse: 5′-TATTGAAGGGGGTTCTGGTG-3′. For analysis of mRNA expression, cDNA was synthesized using the iScript cDNA synthesis kit (BioRad, Hercules, CA, USA). PCR was performed using the iTaq Universal SYBR Green PCR Kit (BioRad). The primers were as follows: CMTM8, forward: 5′-GGAGGAGCCGCAGCGCG-3′, reverse: 5′-CTGTATGGTCCTGGATCTCC-3′; KLF15, forward: 5′-ATGCACAAATGTACTTTCCCT-3′, reverse: 5′-TCAGTTCACGGAGCGCACGGA-3′; FOXG1, forward: 5′-CTTCATCCTGAGTCCCTACCG-3′, reverse: 5′-GCCGTTCTGCTGCATTCG-3′. Relative gene expression was analyzed using the 2^−ΔΔCT^ method [19].

### Oligonucleotides, plasmids, and cell transfection

miR-582-5p mimics (a chemically synthesized RNA duplex; Cat. # miR10003247-1-5), anti-miR-582-5p inhibitor (antisense oligonucleotide; Cat. # miR20003247-1-5), and corresponding controls were purchased from RiBo-Bio (Guangzhou, China). They were used at a final concentration of 50 nM. The miR-582-5p- and CMTM8-expressing plasmids were obtained from Hanyu BioTechnology (Beijing, China). Cells were transfected with the oligonucleotides or plasmids by Lipofectamine 3000 (Invitrogen) as per the manufacturer’s instructions. For assessment of transfection efficiency, cells were transfected with Fluorescent Dye-labeled Pre-miR Negativ Control (Cat. # AM17121, Invitrogen) and analyzed by flow cytometry. In this study, the transfection efficiency reached about 75%. Stable cell lines were prepared by selecting in the presence of G418 or puromycin (Sigma-Aldrich).

### Luciferase reporter assay

Luciferase reporter assay was performed as described previously [20]. The 3′-UTR of *CMTM8, FOXG1*, and *KLF15* was inserted into the pmirGLO dual-luciferase miRNA vector. The QuikChange Site Directed Mutagenesis Kit (Stratagene, La Jolla, CA, USA) was utilized to produce point mutations in the 3′-UTR of *CMTM8, FOXG1*, and *KLF15*. BT-549 cells were co-transfected with the 3′-UTR constructs, and miR-582-5p mimic or control mimic. After 24-h culturing, luciferase activity was measured using the Dual-luciferase Reporter Assay System (Promega, Madison, WI, USA).

### Western blot analysis

Cells were lysed in ice-cold radioimmunoprecipitation assay buffer supplemented with the Protease Inhibitor Cocktail (Sigma-Aldrich). The protein samples were separated by sodium dodecyl sulfate-polyacrylamide gel electrophoresis, and transferred to nitrocellulose membranes. After incubation with primary antibodies recognizing CMTM8 and GAPDH (Proteintech, Wuhan, China), the membranes were probed with peroxidase-conjugated secondary antibodies. Signals were developed by enhanced chemiluminescence (Merck Millipore, Darmstadt, Germany).

### Cell proliferation

Cells were seeded onto 96-well plates and cultured for 1–3 days. The activity of cells were measured using the MTT assay (Sigma-Aldrich). MTT was used at a concentration of 0.5 mg/mL, and after 4-h incubation at 37°C, dimethyl sulfoxide was then added. Absorbance was measured at 490 nm.

### Wound-healing assay

TNBC cells were allowed to grow to confluence. We made a ‘wound’ on the monolayer using a pipette tip. After culturing for 0 and 48 h, the images of the ‘wounded’ monolayer was taken. We then calculated the percentage of wound closure.

### Transwell invasion assay

We used Transwell chambers (8 µm in pore size) to evaluate the invasive ability of cancer cells [14]. Briefly, TNBC cells transfected with indicated constructs were suspended in serum-free media and added onto the upper chamber precoated with Matrigel. The lower chamber was filled with the media containing 10% FBS. After incubation for 48 h, the invaded cells were stained with 0.25% crystal violet and counted.

### Animal experiments

Female BALB/c nude mice (4–5 weeks old) were purchased from Hanyu Biomed Company (Beijing, China). They were maintained in a specific pathogen-free animal facility under a condition of a 12-h dark/light cycle, a temperature of 24 ± 1°C, and a humidity of 52 ± 3%. These animals were given free access to standard rodent diet and water. For tumorigenic studies, stably transfected MDA-MB-231 cell lines were subcutaneously injected into nude mice (2 × 10^6^ cells/mouse). Tumor growth was measured every week for 4 weeks. For metastatic studies, the MDA-MB-231 cell clones were transfected with firefly luciferase-expressing plasmid and injected into nude mice via the tail vein [21]. Six weeks after cell implantation, mice were injected with D-luciferin. *In vivo* bioluminescence imaging was then conducted. In each experiment, 3 groups were included. A total of 12 mice were randomly assigned to the 3 groups, and each group had 4 mice. All the procedures were conducted under anesthesia with intraperitoneal injection of sodium pentobarbital (50 mg/kg body weight; Sigma-Aldrich). After the experiments, anesthetized mice were euthanized through cervical dislocation.

### Statistics

Results were obtained from at least three independent experiments and are expressed as mean ± standard deviation. GraphPad Prism (version 7.0; GraphPad Software, La Jolla, CA, USA) was used to determine statistical differences. The significance was analyzed using the Student’s *t* test or one-way analysis of variance (ANOVA) followed by the Tukey test. A *P*-value less than 0.05 was considered statistically significant.

## Results

### Upregulation of miR-582-5p in TNBC tissues and cells

**Figure 1.**
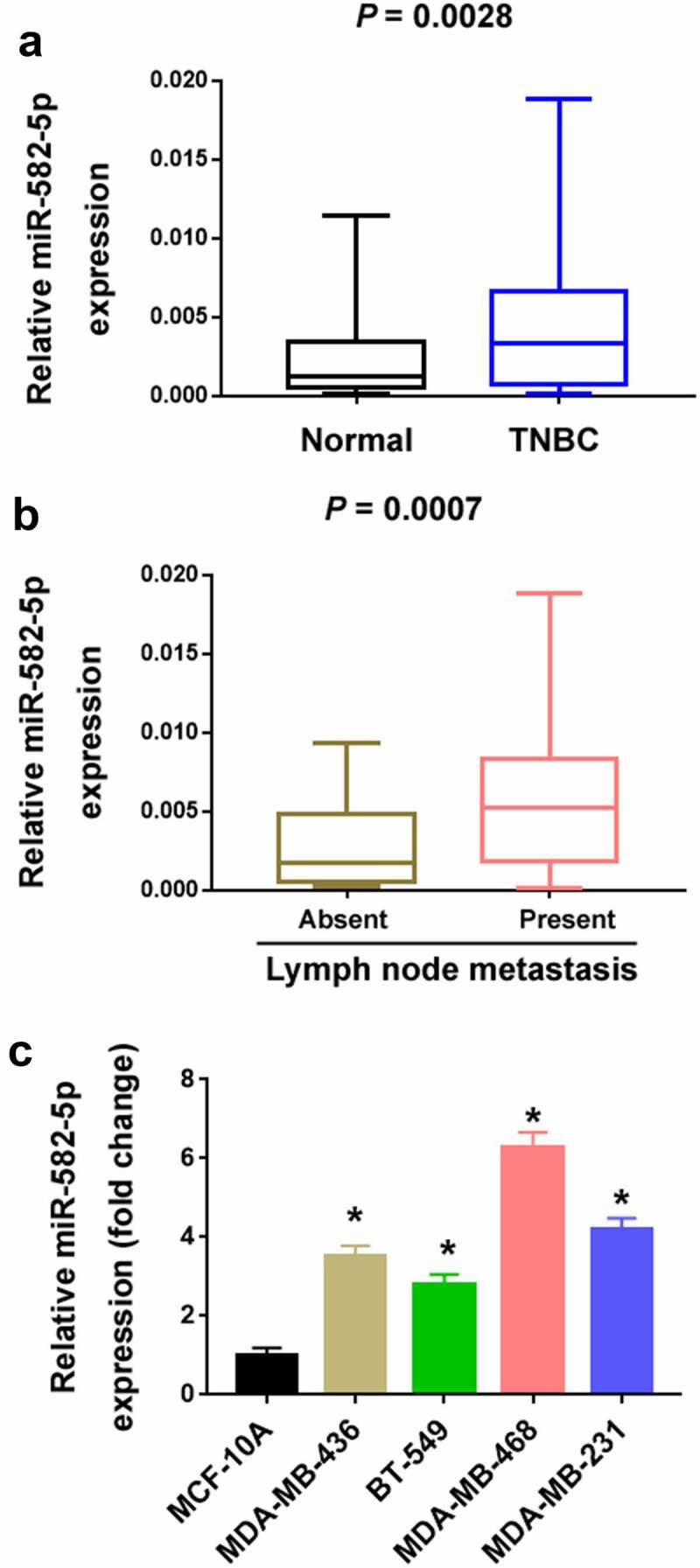
Upregulation of miR-582-5p in TNBC. (a) Quantification of miR-582-5p expression in 68 paired TNBC and adjacent normal tissues. (b) The expression level of miR-582-5p is increased in the tumors with lymph node metastasis (n = 36) compared to those without lymph node metastasis (n = 32). (c) Quantification of miR-582-5p expression in TNBC cell lines and MCF-10A nonmalignant cells. *P < 0.05 compared to MCF-10A

A number of previous studies have indicated the biological roles of miR-582-5p in multiple cancer types [12–18]. In the current study, we attempted to explore its clinical and biological significance in TNBC. We examined the expression of miR-582-5p in 68 TNBC and their corresponding normal breast tissues. The results showed that miR-582-5p was significantly upregulated in TNBC relative to corresponding breast tissues (*P* = 0.0028; Figure 1(a)). Moreover, miR-582-5p overexpression was significantly associated with lymph node metastasis (*P* = 0.0007; Figure 1(b)). Consistent with the clinical findings, TNBC cell lines had higher levels of miR-582-5p than MCF-10A cells (Figure 1(c)).

### miR-582-5p promotes TNBC cell migration and invasion

**Figure 2.**
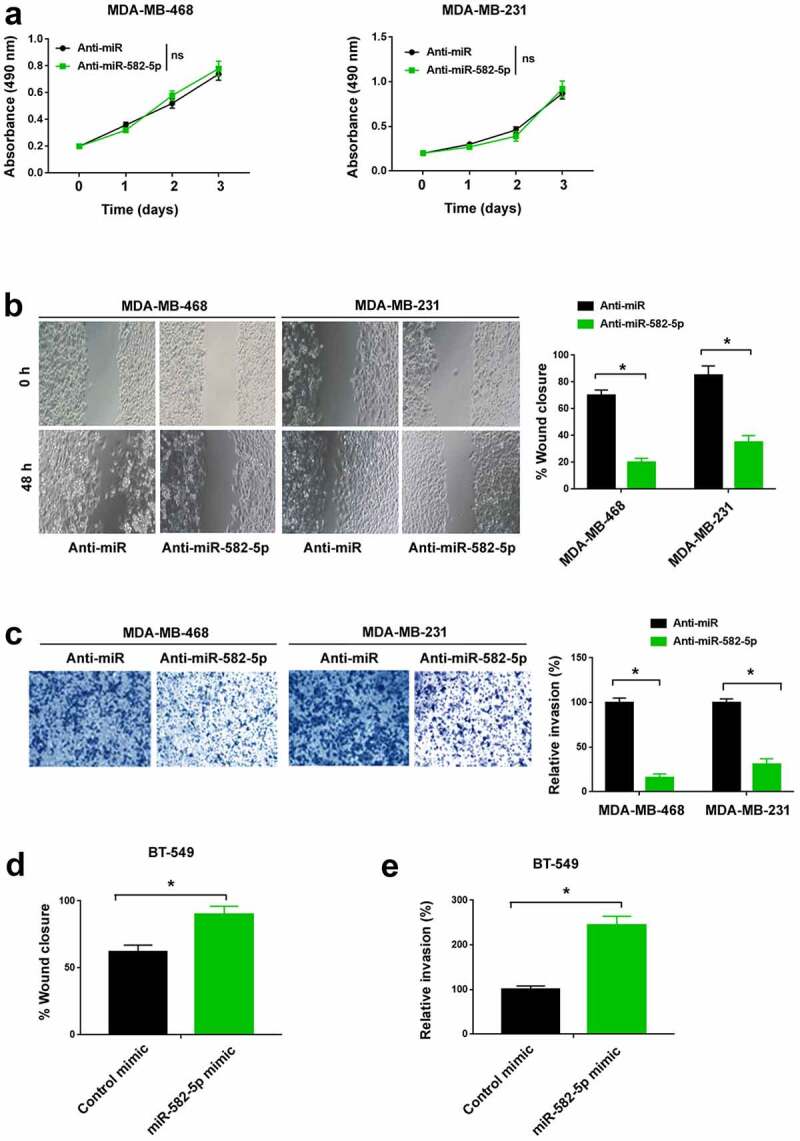
miR-582-5p promotes TNBC cell migration and invasion. (a) MTT assay showed that depletion of miR-582-5p had no effect on the proliferation of MDA-MB-468 and MDA-MB-231 cells. ns indicates no significance. (b) Wound-healing assay. Knockdown of miR-582-5p suppressed cell migration. (c) Transwell invasion assay. The invasion of TNBC cells was reduced by miR-582-5p knockdown. (d,e) Ectopic expression of miR-582-5p promoted the migration (d) and invasion (e) of BT-549 cells. *P < 0.05

To investigate the function of miR-582-5p in TNBC, miR-582-5p was knocked down in MDA-MB-468 and MDA-MB-231 cells by transfecting specific miR-582-5p inhibitors. The proliferation of TNBC cells was not altered by antagonization of miR-582-5p (Figure 2(a)). The wound healing assay demonstrated that knockdown of miR-582-5p significantly decreased TNBC cell migration (Figure 2(b)). Similarly, Transwell invasion assay showed that miR-582-5p deficiency was associated with reduced invasive ability in TNBC cells (Figure 2(c)). Next, we overexpressed miR-582-5p in BT-549 cells. Of note, ectopic expression of miR-582-5p reinforced BT-549 cell migration (Figure 2(d)) and invasion (Figure 2(e)).

### miR-582-5p targets CMTM8 mRNA in TNBC cells

**Figure 3.**
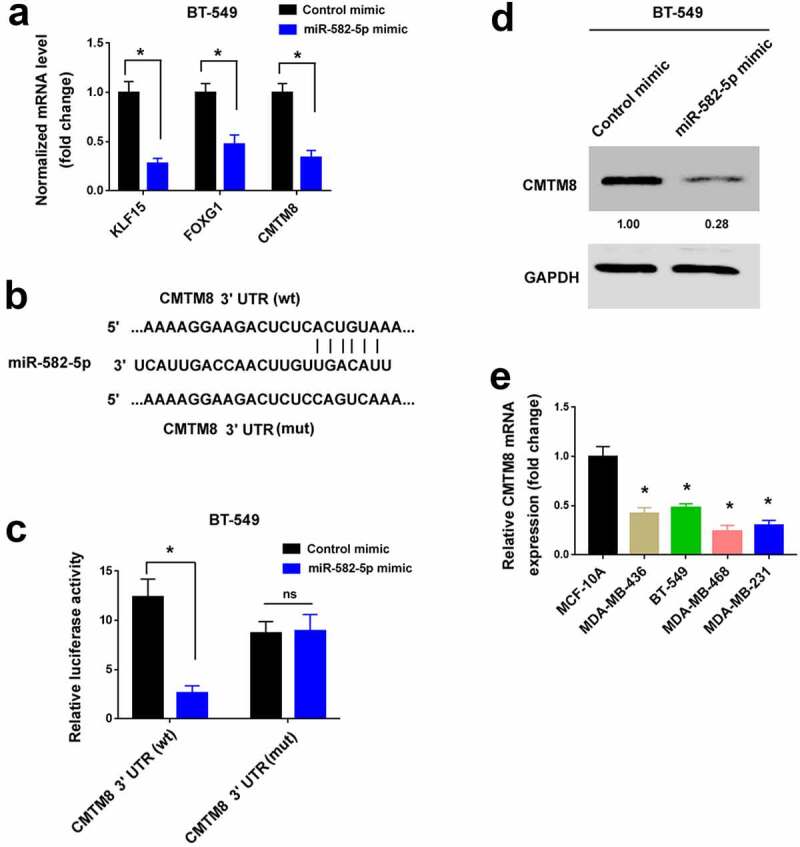
miR-582-5p targets CMTM8 mRNA in TNBC cells. (a) Quantification of KLF15, FOXG1, and CMTM8 mRNA levels in BT-549 cells transfected with control mimic or miR-582-5p mimic. *P < 0.05. (b) Prediction of a putative miR-582-5p target site in the 3′-UTR of CMTM8 mRNA. wt: wild-type; mut: mutant. (c) Luciferase reporter assay demonstrated that overexpression of miR-582-5p suppressed the luciferase reporter carrying the 3′-UTR of CMTM8. *P < 0.05. ns indicates no significance. (d) Western blot analysis of CMTM8 protein levels in BT-549 cells transfected with control mimic or miR-582-5p mimic. (e) Quantification of CMTM8 mRNA levels in TNBC cell lines and MCF-10A nonmalignant cells. *P < 0.05 compared to MCF-10A

TargetScan (http://www.targetscan.org) was used to predict target genes that mediate the pro-invasive capacity of miR-582-5p. We chose 30 candidate genes for further validation by qPCR analysis (data not shown). Among them, KLF15, FOXG1, and CMTM8 mRNA levels were significantly reduced by miR-582-5p overexpression (Figure 3(a)). The other 27 genes remained unchanged after miR-582-5p overexpression (data not shown). We then performed 3′-UTR reporter assays to validate whether miR-582-5p could directly target the 3′-UTR of the candidate mRNAs. The results showed that the luciferase reporter carrying the 3′-UTR of *CMTM8* but not *FOXG1* or *KLF15* was suppressed by overexpression of miR-582-5p (Figure 3(b,c), Supplementary Figure S1). Mutation of the miR-582-5p target sites blocked the repression of *CMTM8* 3′-UTR reporter by miR-582-5p (Figure 3(b,c)). Consistent with the reduction in the CMTM8 transcript level, the protein level of CMTM8 was diminished in miR-582-5p-transfected TNBC cells (Figure 3(d)). We also measured CMTM8 transcripts in TNBC cell lines. Relative to MCF-10A cells, CMTM8 mRNA levels were reduced in TNBC cell lines (Figure 3(e)). These results suggest that miR-582-5p targets the 3′-UTR of CMTM8 mRNA and represses CMTM8 expression.

### CMTM8 is involved in miR-582-5p-mediated invasiveness of TNBC cells

**Figure 4.**
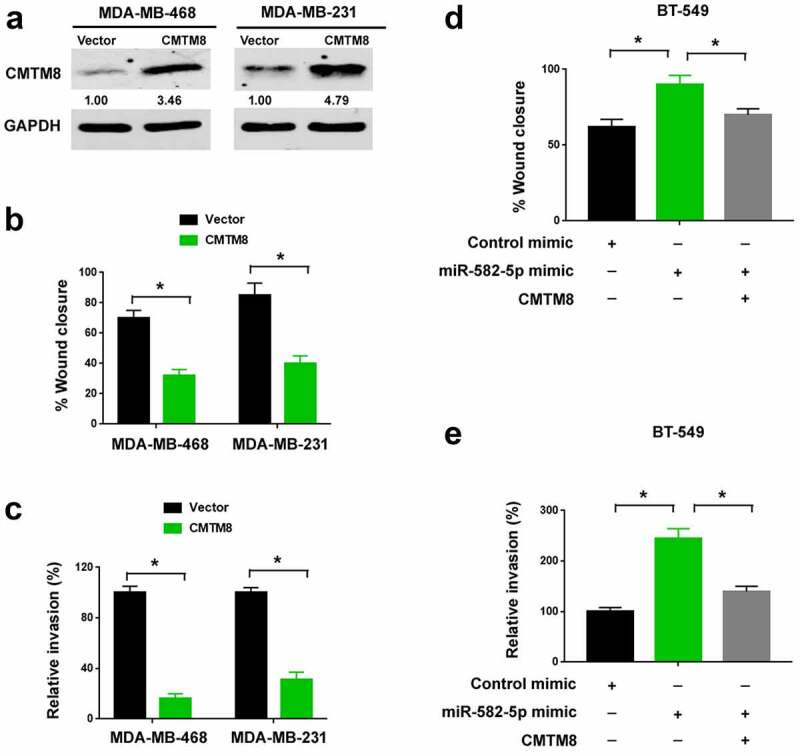
CMTM8 is involved in miR-582-5p-mediated invasiveness of TNBC cells. (a) Western blot analysis of CMTM8 protein levels in TNBC cells transfected with empty vector or CMTM8-expressing plasmid. (b,c) Ectopic expression of CMTM8 suppressed the migration (b) and invasion (c) of MDA-MB-468 and MDA-MB-231 cells. (d,e) BT-549 cells were transfected with indicated constructs and subjected to wound-healing (d) and Transwell invasion (e) assays. *P < 0.05

Next, we asked whether CMTM8 has an impact on TNBC progression. To this end, we overexpressed CMTM8 in MDA-MB-468 and MDA-MB-231 cells (Figure 4(a)). We found that the migratory (Figure 4(b)) and invasive (Figure 4(c)) abilities of TNBC cells were suppressed by enforced expression of CMTM8. Also, we tested whether CMTM8 is involved in the pro-invasive activity of miR-582-5p. To address this, we performed rescue experiments in miR-582-5p-overexpressing TNBC cells. Notably, restoration of CMTM8 expression significantly impaired miR-582-5p-dependent migration (Figure 4(d)) and invasion (Figure 4(e)) in TNBC cells. Taken together, the pro-invasive role of miR-582-5p is causally linked to downregulation of CMTM8.

### miR-582-5p enhances TNBC metastasis by targeting CMTM8

**Figure 5.**
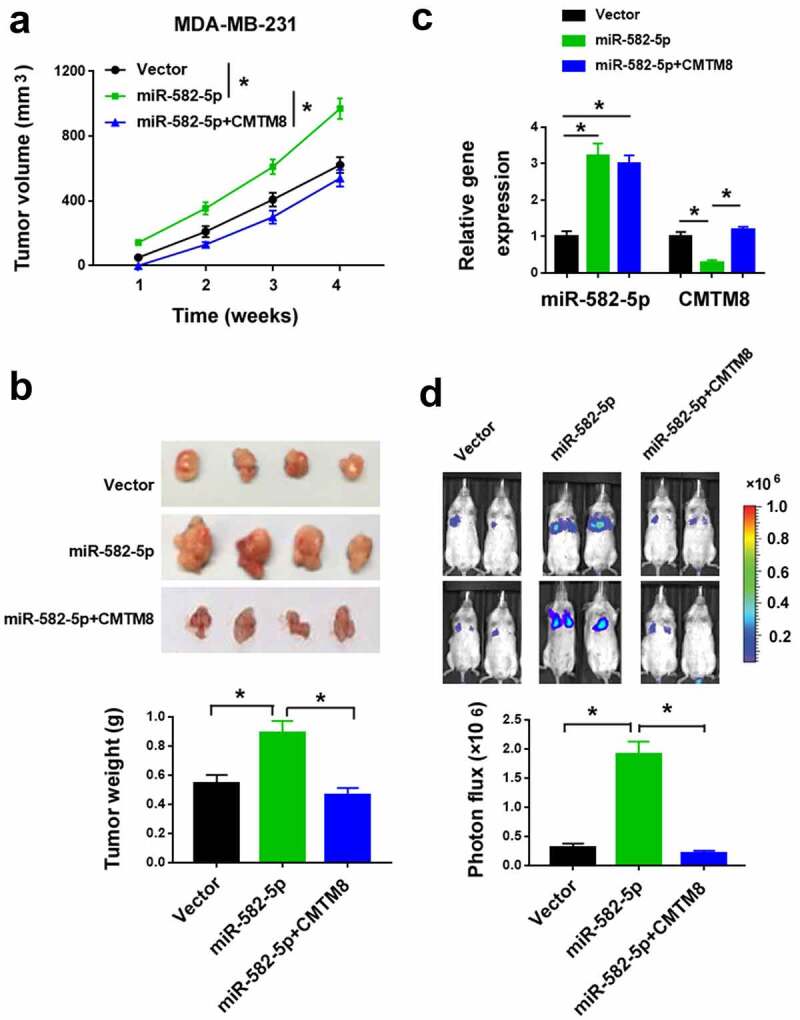
miR-582-5p enhances TNBC growth and metastasis in vivo by targeting CMTM8. (a) Tumor volumes were measured in nude mice subcutaneously injected with MDA-MB-231 cells transfected with indicated constructs (n = 4 for each group). (b) Top: representative images of the xenograft tumors. Bottom: tumor weight was determined 4 weeks after cell injection. (c) Quantification of miR-582-5p and CMTM8 mRNA expression in the xenograft tumors. (d) Top: representative images of bioluminescence imaging of mice 6 weeks after intravascular injection of indicated cells. Bottom: statistical analysis of photon flux (n = 4). *P < 0.05

We also validated the activity of miR-582-5p in animal models. We injected parental and miR-582-5p-overexpressing MDA-MB-231 cells into the mammary fat pad of SCID mice. The miR-582-5p overexpression group had an increased tumor growth than the control group (Figure 5(a-b)). qPCR analysis confirmed the increased expression of miR-582-5p and decreased expression of CMTM8 mRNA in miR-582-5p tumors compared to control tumors (Figure 5(c)). In addition, miR-582-5p overexpression increased the metastatic burden in the lung (Figure 5(d)). Most importantly, co-expression of CMTM8 significantly reversed the effects of miR-582-5p on metastasis (Figure 5).

## Discussion

TNBC is an aggressive type of breast cancer and lacks effective therapies [1,22]. Identification of key regulators of TNBC progression is of importance for developing novel anticancer strategies. Many miRs have been found to modulate TNBC growth and metastasis [23,24]. For instance, miR-92a-3p overexpression enhances breast cancer cell proliferation and metastasis [23]. Ectopic expression of miR-210-3p stimulates aerobic glycolysis and promotes TNBC cell survival and growth under metabolic stress conditions [24]. In the present study, we show that miR-582-5p plays an oncogenic role in TNBC. Compared to adjacent normal breast tissues, TNBC specimens have greater levels of miR-582-5p. The upregulation of miR-582-5p is associated with lymph node metastasis of TNBC. However, there is no significant association between miR-582-5p expression and tumor size or stage (data not shown). In future work, we will validate the clinical significance of miR-582-5p expression in an independent, larger cohort of breast cancer patients. Functional studies reveal that miR-582-5p promotes TNBC cell migration and metastasis. These findings suggest miR-582-5p as a novel driver of TNBC metastasis.

Although previous studies have established a link between miR-582-5p and tumor progression [12,14,17,18], its biological effects dramatically vary among different cancer types. For instance, when miR-582-5p is overexpressed, bladder cancer [12] and non-small cell lung cancer [22] had a reduced proliferation and invasion. Conversely, in glioblastoma, miR-582-5p enhances cancer stem cell survival [17]. Under androgen deprived conditions, miR-582-5p augments prostate cancer cell proliferation [18]. Our data also show the oncogenic role of miR-582-5p in TNBC. Depletion of miR-582-5p impairs the migration and invasion of TNBC cells. Moreover, enforced expression of miR-582-5p promotes TNBC cell invasion and metastasis. However, TNBC cell proliferation remains unchanged when miR-582-5p is silenced. Our results support the pro-invasive activity of miR-582-5p in TNBC.

NOTCH1, AKT3, and CDK1 [13,15,25] have been proposed as the target genes of miR-582-5p. In hepatocellular carcinoma, miR-582-5p exerts growth-suppressive effects through negative regulation of CDK1 and AKT3 [13]. In prostate cancer, miR-582-5p can inhibit tumor metastasis by targeting several components of TGF-β signaling, including SMAD2, SMAD4, TGFBRI, and TGFBRII [16]. However, miR-582-5p did not affect these gene expression in TNBC cells (data not shown). Most interestingly, we show that miR-582-5p targets CMTM8 mRNA, leading to a reduction in the abundance of CMTM8 mRNA. Luciferase reporter assay confirms that miR-582-5p-mediated repression of CMTM8 is ascribed to the interaction with the target site in the 3′-UTR of CMTM8 mRNA. Furthermore, the expression of miR-582-5p and CMTM8 mRNA shows an opposite trend in TNBC cell lines. In addition to CMTM8, both KLF15 and FOXG1 are also downregulated by miR-582-5p overexpression. The 2 genes seem not to be miR-582-5p targets, as the luciferase reporters carrying their 3′-UTR remained unaffected when miR-582-5p was overexpressed. Collectively, miR-582-5p selectively targets CMTM8 mRNA in TNBC cells.

It has been previously documented that CMTM8 expression is reduced in several cancer types such as liver, lung, colon, rectum, esophagus, stomach cancers [5]. However, it is still unclear how CMTM8 is downregulated in malignant tissues. In this study, we demonstrate that miR-582-5p overexpression suppresses CMTM8 expression in TNBC cells, suggesting a mechanism for the downregulation of CMTM8 in cancer. CMTM8 has been reported to inhibit the proliferation and invasion of bladder cancer cells [7]. In agreement with this study, we show that restoration of CMTM8 decreases the migratory and invasive abilities of TNBC cells. Furthermore, enforced expression of CMTM8 impairs miR-582-5p-induced TNBC cell invasion and metastasis. These results suggest that miR-582-5p promotes TNBC metastasis by targeting CMTM8. However, we can not rule out the possibility that other target genes may also mediate the oncogenic activity of miR-582-5p.

## Conclusion

We identify miR-582-5p as a novel oncogene in TNBC. MiR-582-5p promotes TNBC invasion and metastasis by negatively regulating CMTM8 expression. We propose miR-582-5p as a potential therapeutic target for TNBC.

## Supplementary Material

Supplemental Material

## Data Availability

The datasets during and/or analyzed during the current study available from the corresponding author on reasonable request.
