# Supplementary material for: MicroRNA-582-5p promotes triple-negative breast cancer invasion and metastasis by antagonizing CMTM8
Source: Bioengineered. 2021 Dec 2;12(2):10126–35. doi: 10.1080/21655979.2021.2000741 (PMC8810067; doi:10.1080/21655979.2021.2000741)
Supplement: Supplemental Material [file KBIE_A_2000741_SM6746.doc]

**Supplementary data**


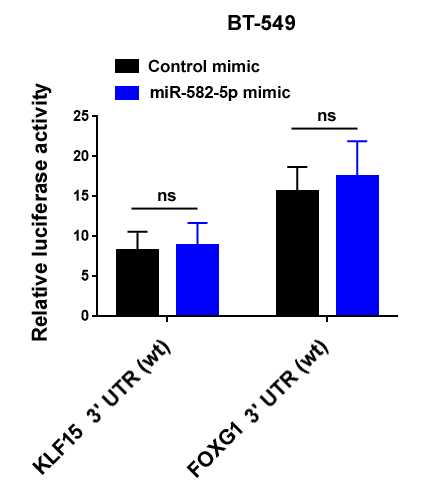


Supplementary Figure S1. Luciferase reporter assay showed that overexpression of miR-582-5p did not affect the luciferase reporter carrying the 3′-UTR of KLF15 or FOXG1*.* ns indicates no significance. wt: wild type.


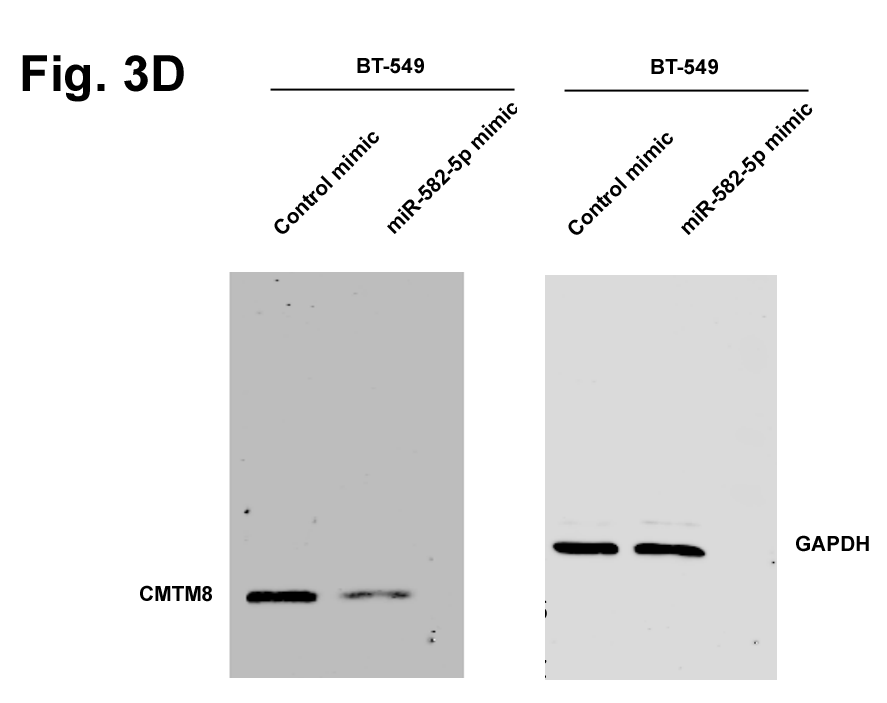

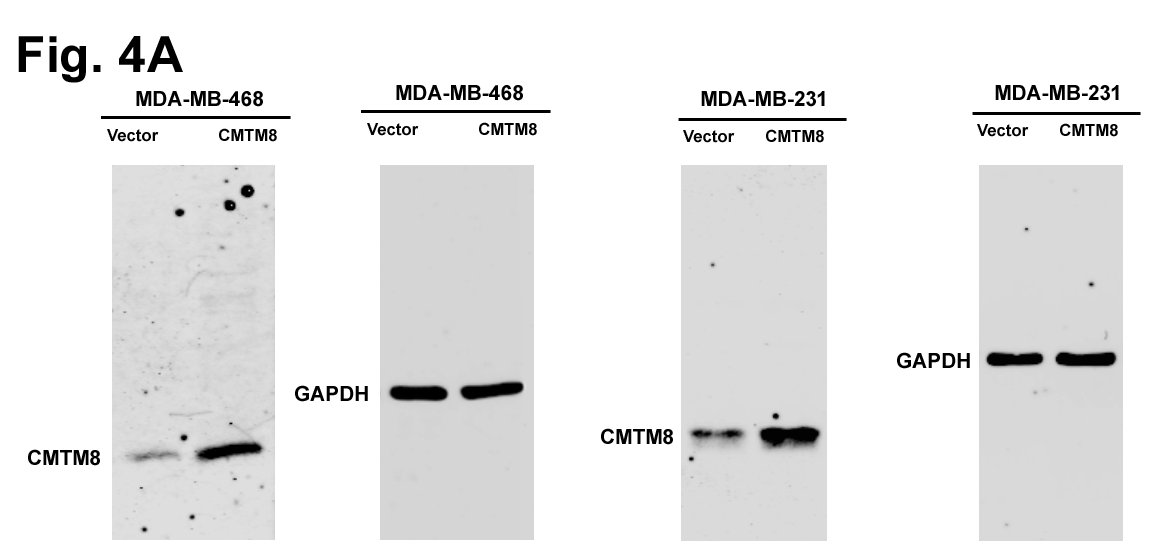


Full scans of Western blots.
